# Alzheimer’s Disease Research and Outreach in the New Normal: Transitioning to the Virtual World

**DOI:** 10.1093/geroni/igab046.525

**Published:** 2021-12-17

**Authors:** Stacy Andersen, Patricia Heyn

## Abstract

Following disruptions to research, clinical trials, and support for individuals with Alzheimer’s disease and related dementias (ADRD), 2020 taught us important lessons about the need for creativity, flexibility, and resourcefulness during an urgent and global public health crisis. The COVID-19 pandemic showed that we have knowledge and technology that can be rapidly implemented, adopted, and utilized by many sectors to allow the continued care and research of our older adult population with ADRD. Thus, this symposium will address virtual methods that are transforming ADRD research and support. First, Dr. Rhodus will discuss the implementation of online assessments in clinical trials at an Alzheimer’s Disease Research Center and the effects of sociodemographic disparities in online accessibility. Next, Dr. Bazzano will describe methods of remote collection of brain health data through tablets, smartphones, and wearables in the Bogalusa Heart Study. Then, Dr. Andersen will report on the transition from in-person to virtual assessments of cognitive and physical function in centenarian studies and address strategies for inclusivity of individuals with limited technology experience. Next, Dr. Fazio will introduce Project VITAL which aims to impact social isolation by increasing accessibility to virtual education and support for care community staff, family caregivers, and individuals with dementia. Finally, Dr. Penfold will report on the translation of a paper-based, face-to-face intervention for reducing caregiver burden into a self-directed online learning program. Overall, these presentations highlight successes and challenges in incorporating virtual-based methods to maintain engagement with participants, individuals with ADRD, and caregivers during the pandemic and beyond.

